# Social media as a tool to monitor adherence to HIV antiretroviral therapy

**Published:** 2018-03-20

**Authors:** David Bychkov, Sean Young

**Affiliations:** ^1^ *Johns Hopkins University, Whiting School of Engineering, Institute for NanoBioTechnology,inHealth Measurement Corps, Baltimore, Maryland, USA*; ^2^ *Institute for Prediction Technology, Department of Family Medicine, University of California, Los Angeles, California, USAUniversity of California, Los Angeles, California, USA*

**Keywords:** intestinal failure, liver disease, enterohepatic cycle, bile salt signaling, FXR, enterocutaneous fistula

Although strict adherence to antiretroviral therapy (ART) lowers morbidity and mortality for people living with HIV and AIDS, there are a number of behavioral, cultural, and psychosocial factors that prevent people from adhering to their medication [1] . Within African -American and Latino communities, for example, stigma around sexual identity and behavior hinders open dialogue about the value and proper use of HIV treatment medications [2,3]. In addition, misunderstandings about how ART affects the body have led some patients to believe that they can skip doses without experiencing negative consequences. For example, in 2015, the actor Charlie Sheen—who has been influential in raising public awareness about HIV—announced on *The Dr. Oz Show* that he has strayed from his ART regimen [4]. As a result, public health researchers require innovative methods for monitoring and improving adherence to HIV and other medications.

A number of factors affect medication adherence, including potential stigmatization from taking medication or being associated with testing positive for HIV [5, 6]. As patients are publicly sharing personal information, including HIV related behaviors, on social media [[7,[8[9], health systems may be able to gain insights into patients’ adherence by gaining consent from patients to follow and monitor their social media accounts. Health systems could use that information to assist with health care decisions. However, many patients and health systems might be uncomfortable with the potential legal and ethical issues associated with tracking individual patients. Therefore, it’s also possible to avoid directly following individuals and instead use patients’ publicly-available aggregated data to understand and predict adherence-related behaviors. For example, hospital patients are actively using Twitter to ask questions and express concerns about their healthcare experiences [10], and this information can be mined to improve patient outcomes.

People also use social media to express their emotions and stress levels, including anger, fear, happiness, and others [11]. This psychological information has been used as an indicator for mental health conditions such as depression [12] and post-traumatic stress disorder [13]. Mining this psychological information about patients could give care providers a real-time sense of patients’ emotional states and possible insight into reasons for medication adherence. If provided with this information, hospitals may be able to respond to the social media concerns of HIV-positive patients, who are already at a higher risk for medical errors and hospital readmission [14], in order to help improve adherence and clinical outcomes among these patients.

Most American Hospital Association–member hospitals maintain an active Twitter account [15], although accounts are often limited to releasing press releases or general health information. Hospitals might therefore integrate Twitter’s free, open access architecture to gather more information about adverse medical events and tailor public awareness campaigns to combat misconceptions about ART. Twitter can be used to aggregate data and maintain anonymity, while offering optional geo-tagging that would enable healthcare providers to track communications and develop targeted responses.

Discussions about HIV medications can also be mined on social media. Manufacturers of the most commonly prescribed ART drugs, such as Truvada and Atripla, have dedicated Twitter handles that can be studied. A Twitter search using the hashtag “#truvada” returned 3,350 tweets from users discussing fears, side effects, and missed doses of the drug. Another search using the term “poz meds” returned 283 tweets, including several that describe misuse or lack of adherence to ART medications. [“Poz” is a term used by HIV-positive individuals who openly discuss their status.] A third search, for the phrase “HIV meds side effects,” yielded 262 tweets, including some where users described specific details of their drug usage (Table 1).

**Table 1.** Examples of tweets related to ART medication use/misuse*

Twitter search Term	Example tweet
“#truvada”	x201C;I've been trying to swallow #Truvada all year. Here's an essay on medicine, community & fear describing 21 attempts: http://www.slate.com/blogs/outward/2014/12/05/truvada_the_gay_community_and_fear_considering_the_meanings _of_condoms_and.html (December 5, 2015) x201C;This is why I'll never take #prep #truvada Longtime #hiv survivors are also having long term use of med probs!https://twitter.com/hivequal/status/688039025962266627” (January 15, 2016) x201C;Gr. Forgotten meds past 2 eves and taken in morning... Had beans for breakfast #whatcouldgowrong #truvada #HIV”(January 5, 2015) x201C;It was all fine all this while. But today, Day 5 on #PrEP #truvada, is when the nausea hits Ugh. Not fun #gayboyproblems”(April 14, 2016)
“poz meds”	x201C;Guys on Grindr wanting me to stop taking my meds so I can 'poz them up'.” (October 1, 2016) x201C;Stopped taking meds now for few months and feel way more horny and sleazy! Who else is the same while off meds? #bbbh #poz” (September 28, 2016) x201C;@Rx_Ed I'm Poz. Sometimes go on personal breaks from HIV meds w/o docs knowledge/approval. Many friends in same boat. Thoughts?”(August 15, 2016)
“HIV meds	x201C;Yall ever read the side effects that hiv meds have?? Wtf #theseWHITEPEOPLEKillinyall #NoSuchThingAsHIV #KNOWyaSTATUSpic.twitter. side effects” com/9ncKemSlKE.” (September 12, 2015) x201C;Kind of scared to take my HIV meds, my body is on west coast time and since I have trouble sleeping I might not sleep off the side effects!”(November 30, 2010) x201C;Began my HIV meds last night it actually wasnt as bad as i thought only side effects were hot flashes and acute naseau.” (October 4, 2009) x201C;@aidsmap I take 1 pill once a day, another pill twice a day. Alarm on phone reminds me. No side effects. Had #HIV for 12 yrs; on meds 3yrs” (May 14, 2014)

“Poz” is a term used by HIV-positive individuals who openly discuss their status.

The question then becomes: How could hospitals integrate Twitter data into their clinical monitoring and care for HIV patients? As a guide, they can look to how primary care doctors handle patient comments on other social media platforms like Yelp and ZocDoc. Small business owners in the healthcare sector, and nearly all customer-oriented industries, monitor social media conversations and use these popular tools to communicate with customers and patients as users of the system [16, 17]. On ZocDoc, an appointment-setting service, patients are encouraged to post reviews of their medical care experiences. Patients have an opportunity to rate their provider’s bedside manner, waiting time, and overall experience. In addition, patients frequently comment on treatment plans, whether their questions were answered, if they felt rushed, or if there was a medical error that occurred during their visit [18]. Because these ratings impact the financial performance of individual practitioners, clinicians are incentivized to address the concerns of patients in the ZocDoc blog section and offline.

Readmissions and preventable visits caused by poor adherence to ART provide a similar financial incentive to hospitals. The typical ART regimen consists of a combination of HIV medications, frequent viral load and CD4 count testing, and a healthy diet and lifestyle. ART medications may be accompanied by side effects, such as nausea, diarrhea, headache, or mood swings. In fact, gastrointestinal symptoms were identified as the primary reason for discontinuation in 28.5% of cases in a cohort study of 1,096 patients [19]. Other reasons patients discontinue ART include strong concerns about HIV stigma that can lead them to misinterpret medical information about side effects. These “stigma-sensitive” patients are 2.5 times less likely to correctly interpret the meaning of their CD4 count, and 3.3 times more likely to stop adherence to an ART regimen than patients who identify as having minimal concerns [20]. In response to this, a social media-based monitoring system would enable patients who have stigma concerns to anonymously communicate with hospital personnel about sexual health, medication side effects, and counseling needs via Twitter.

Training hospital staff and patients to use dedicated hashtags and handles that can be used in case of adverse events, missed doses, confusion about lab results, and/or misunderstanding about ART instructions would reduce time spent on false reporting and parsing irrelevant data. Naturally, hospitals would have to invest in staff and technologies such as automated or customized replies to patients who send emergency requests for assistance about their medication. Fortunately, best practices are already in place from first responders who have used Twitter to communicate with people in emergency situations, such as stranded motorists [21]. Although data privacy issues would need to be resolved before implementing this strategy—including expectations regarding data reach and permanence; individual attitudes toward data monitoring; and attitudes about monitoring a stigmatized condition such as HIV—hospitals could take the first step of asking ART patients for their Twitter handles prior to discharge [22]. In addition, patients would need to provide consent to monitor their off-site tweets, and care teams would ask patients to provide daily updates to followers on how their ART regimen is progressing.

Overall, hospitals have already invested substantially in electronic medical records and social media marketing campaigns. The opportunity is to now leverage those two investments to capture and store relevant data about HIV patients on ART regimens, all the while ensuring privacy concerns are met. The good news is Twitter’s open application programming interface enables clinicians to monitor user activities, including the frequency and time of tweets. If a patient were to tweet specific words related to their treatment, such as “Truvada,” “nausea,” “pain,” or “stomach,” the data could become part of their electronic health record and trigger heightened surveillance measures. In many cases, an alert that a patient is confused or distressed about their ART treatment might be enough to warrant early intervention. In turn, it may be possible to reduce the overall time and costs of treating HIV-positive patients. Due to the sensitivity around HIV, additional research should be conducted to determine the ethical issues associated with this approach and how to address them.
